# His Majesty's Practical Interest in Science

**Published:** 1910-05-14

**Authors:** 


### KING EDWARD VII. AND MEDICINE.

### HIS MAJESTY'S PRACTICAL INTEREST IN SCIENCE.

The name of King Edward VII. is not to be found in the Medical Register, nor, except in so far as it heads the list of the medical officers of the Royal household, in the Directory. For all that his late Majesty, although he was not a medical man by examination and training, was entitled to a place in both these volumes, since he was an honorary member of the profession, and one, moreover, who always took a keen personal interest in all that concerned the advance of medicine within his realm and outside of it. He was the Royal patron of many learned societies, and one of the most recent extensions of his patronage was when he graciously signified to the Glasgow College his pleasure that the old and famous corporation should henceforward be known as the Royal College of Surgeons. Since 1897 he has been an honorary Fellow of the Royal College of Physicians of London, and he has been patron of nearly all the Universities which have a medical faculty. Of late years his support and patronage have been especially valuable to the newer schools, such as those of Manchester, Leeds, and Sheffield, and his well-known interest in the various schools of tropical medicine showed that he was fully alive to the importance of medical research. He was always ready to recognise merit in the medical profession, and rewarded it suitably and generously. His recent action in honouring Professor Kutner was a graceful act that will be appreciated by everyone interested in the advancement of post-graduate study in medicine, but it is worth while to bear in mind that, above all, he was sensible of the good work that members of the profession who were his own subjects were doing. During his short reign no less than ten medical baronets were created, a record, so far as time is concerned, which is held by no other Sovereign. In addition he founded the Order of Merit and recognised the sterling achievements of two members of the profession, whose names are household words, by creating them foundation members of the Order. Sir Joseph Hooker's name, it is true, is better known to botanists than it is to medical men, but Lord Lister's is one that will be cherished and honoured so long as modern medicine lasts, and his Majesty's action in so strikingly endorsing, by conferring the Order of Merit upon the great surgeon, the good work that the latter has done was highly appreciated by the profession. It would be difficult to say offhand how generously, even lavishly, his late Majesty has distributed honours among the members of the profession, but few will deny that in every case where a knighthood or an order was conferred the recipient had done much to merit the distinction awarded him. In many cases it is well known that his late Majesty's personal interest had much to do in securing to earnest and little-known workers in the field of medicine a well-deserved acknowledgment of their eminence as scientists.

### His Interest in Medicine.

His Majesty's stimulating influence upon medicine and science, as upon art and social questions, undoubtedly dates from the memorable speech which he delivered on the occasion of his first public appearance at the Academy dinner a few months after his marriage. He there declared that he hoped to follow in the footsteps of his father, the late Prince Consort, and would do his utmost to encourage all that his father had encouraged. Subsequently he showed on numerous occasions how practical and thorough was the interest he took in medical work. His influence upon the hospitals is dealt with in another article, but it should be remembered in connection with his influence upon medical thought and evolution in this country that the late King, as Prince of Wales, was keenly interested in modern hospital construction, and paid great attention to the plans for the building of what was then considered to be the most up-to-date and efficiently constructed hospital in the United Kingdom, the magnificent series of buildings designed on the river for St. Thomas's Hospital. He attended the opening of the new hospital by the Queen in 1871, and in July of the same year his Royal Highness, in his official capacity as President of St. Bartholomew's Hospital, paid a visit to that institution to present to Sir (then Mr.) James Paget, on behalf of the Court of Governors, an illuminated copy of a resolution passed by the Court to express its regret at the senior surgeon's retirement. During the same year his Royal Highness paid various visits to other hospitals and medical institutions, but his illness in the latter part of the year drew him closer towards the profession. That illness was anxiously and earnestly watched by the profession, as well as by the public at large, and the medical papers of the day abound with comments upon it. It was, for instance, known that the Prince had some bronchial trouble following the attack of typhoid fever, and the chances of tubercular mischief were gravely discussed in one paper. Another complication which was rumoured to exist was suppuration about the hip joint, but all these pessimistic prognostications remained unfulfilled, and his Royal Highness recovered and went on his Continental tour.

### Scientific Research.

On his return he threw himself even more earnestly into hospital and medical work. He presided over festival dinners, opened hospitals and convalescent homes, visited asylums, and showed that he was keenly interested in the developments in the institutional and scientific world. From boyhood, when he had attended Faraday's lectures, the King had displayed an active interest in science, and after his illness he recognised, more perhaps than he had previously done, the outstanding importance of developing and encouraging medicine in its various branches. He was always in favour of educating the public in things medical, and was one of the first to welcome and cordially support the now deservedly popular lectures of the St. John Ambulance Association, which were first started in 1888. He readily consented to become President of the movement, and attended the great gathering at Middlesborough, where he presented the first certificates. His practical interest did not stop here, for he made himself thoroughly familiar with the course of instruction and first-aid.

### The International Medical Congress.

The year 1881 saw the International Medical Congress gathered for the first time together in London, and the Prince of Wales attended its opening meeting and accepted the office of patron. His speech at the first meeting of the Congress showed how sincerely he welcomed this international discussion, and how much he hoped from it. His hopes have in part been realised. Older readers will remember that one of the burning questions at the Congress was the admission of women, and that the Committee finally decided that no women should be allowed to attend, although they had been permitted and encouraged to join the Congress at other centres. This example of prejudice and foolish conservatism was not commented upon by the Prince, but it is reasonable to suppose that he was not in favour of the exclusion of women practitioners, since he has always welcomed the advances made by lady scientists, and his admiration for the work of Madame Curie has been commented upon in the French Press. The next meeting of the International Congress of Medicine at London will miss his patronage and his support, and it is lamentable that his Majesty has not been spared to welcome as King the Association that he so cordially greeted as Prince of Wales.

### The Prevention of Consumption.

During his reign King Edward has shown his friendship towards the profession and his appreciation of the benefits conferred by modern medicine in many and various ways. After his recent severe illness his interest in the surgical side of modern medical science was largely increased. With Sir Frederick Treves he visited the Royal College of Surgeons and inspected the exhibits, spending more than one afternoon in interestedly walking the galleries. As has already been said he took as active an interest in the great questions of the day. The prevention of consumption and the establishment of consumption sanatoria was a matter that specially interested him, while the erection of orphan asylums and institutions was another of his chief interests. For scientific medicine, as such, his interest was, perhaps, less pronounced, yet his support to the tropical investigations, the sleeping sickness bureau, and the appeals for aid to the medical schools and the erection of new and up-to-date laboratories is well known. His prompt action in founding the King Edward's Sanatorium, so admirably equipped, drew the public attention prominently to the urgent necessity of providing new and up-to-date institutions for the investigation of disease. The interest he has shown in new methods of treatment and recent advances in medical science was always practical, and it must be borne in mind that while Queen Alexandra by her generous gift to the London Hospital practically inaugurated the actino-therapeutic department in hospitals, his late Majesty, by his support of the Radium Institute, gave as great a stimulus to one of the newer methods of combating disease. Nor was his late Majesty's attention confined purely to medical science, though it is undoubtedly a fact that in this special department of knowledge his influence has been more felt than in other branches. He was a member of the Institute of Engineers, and followed the progress that was made in this country and abroad in sanitary, electrical, and civil engineering. On various occasions he had the opportunity of closely inspecting modern marvels of engineering science, and displayed a wide knowledge of technical details which showed that his interest was not merely that of a dilettante amateur. Many such occasions will be called to mind by readers who have followed his career, and it is astonishing enough to the medical man, who finds it so difficult to keep pace with the developments in one branch of science only, to note the fact that his late Majesty found time and inclination to become acquainted not merely superficially, but in many cases very thoroughly, with the essentials in so many and diverse sciences.

### The King and the Profession.

His reign has seen, in England at least, no marked and epoch-making advance in medicine as, for instance, Queen Victoria's reign saw in the introduction of antiseptic surgery. The vigorous development of the crusade against consumption and the marked advance in the region of serum therapy with which in England the name of Wright is inseparably connected, are perhaps the chief events with which the medical historian of the future will have to deal. The establishment of the Royal Society of Medicine must also be adduced, while the admission of women to the colleges, and indeed the general broadening of the sphere of women's work in medicine, may be referred to with equal truth as forward steps in the march of progress in England.

In his personal relations with the profession his Majesty was courteous and friendly in the extreme. He showed that he valued and appreciated the work of those whom he honoured, and his personal medical entourage will feel his loss very deeply. He chose his medical attendants with care and discrimination, and their names are those of men who are universally honoured in the profession and whose professional standing is unquestioned. The importance of bacteriological science his late Majesty recognised and acknowledged by creating a special post, that of bacteriologist to the royal household, a post which is now held by Dr. Spitta. In England his physicians-in-ordinary were Sir Francis Laking, Sir James Reid, and Sir Douglas Powell; his physicians extra-ordinary, Sir Felix Semon, Dr. Dawson, and Sir Alan Manby; the physician to the household, Sir Thomas Barlow; the Sergeant Surgeons, Lord Lister and Sir Frederick Treves; the Honorary Surgeons, Mr. Thomas Bryant, Sir Alfred Fripp, and Mr. Godlee; with Mr. Bowlby as Surgeon to the Household, Sir Anderson Critchett as Surgeon Oculist, Sir Henry Longhurst as Surgeon Dentist, and Dr. Hewitt as Anæsthetist. In Scotland his medical staff included Sir Thomas Fraser, Dr. Finlay, and Sir William Macewen, Professor Ogston, Dr. Berry and Dr. Alexander; and in Ireland Sir Francis Cruise and Dr. Little, Sir Charles Ball and Dr. Fitzgerald.

